# Mindfulness Profiles and Substance Use Outcomes in University Students: The Role of Alcohol and Cannabis Use Motives

**DOI:** 10.1007/s12671-025-02544-5

**Published:** 2025-02-28

**Authors:** Folly Folivi, Adrian J. Bravo, Matthew R. Pearson

**Affiliations:** 1 Department of Psychological Sciences, William & Mary, Williamsburg, VA USA; 2 Center on Alcohol, Substance Use, and Addictions, University of New Mexico, Albuquerque, NM USA

**Keywords:** Mindfulness, Substance use, Latent profile analysis, University students, Substance use motives

## Abstract

**Objectives:**

The present study aimed to identify distinct profiles of mindfulness among a sample of university students in the USA who use alcohol and cannabis. Further, we examined whether these mindfulness profiles were indirectly associated with alcohol and cannabis-related outcomes via alcohol and cannabis use motives.

**Method:**

Latent profile analysis (LPA) was used to determine the number of latent classes among 771 US university students (75.7% White, 66.8% female) who consumed alcohol and cannabis in the prior month. Additionally, parallel mediation analyses were conducted to determine whether mindfulness profiles were indirectly associated with alcohol- and cannabis-related outcomes via alcohol and cannabis use motives.

**Results:**

LPA indicated a 4-class solution fit optimally. Further, the high mindfulness group was generally the most adaptive (lower scores) across alcohol and cannabis outcomes, whereas the judgmentally observing group was generally the most maladaptive (higher scores). Indirect effect analyses revealed that compared to the low mindfulness group, the high mindfulness group reported lower scores on alcohol- and cannabis-related outcomes via lower alcohol- and cannabis-related coping motives.

**Conclusions:**

These findings can inform prevention and intervention efforts using mindfulness techniques and interventions among students who engage in problematic alcohol and cannabis use.

**Preregistration:**

This study is not preregistered.

**Supplementary Information:**

The online version contains supplementary material available at 10.1007/s12671-025-02544-5.

Substance use among university students is a global health concern that has increased in recent decades (Kerr & Bae, 2025; Welsh et al., 2019). Importantly, alcohol and cannabis continue to be the most prevalent substances used on university campuses today (Johnston et al., 2022; National Institute on Alcohol Abuse and Alcoholism [NIAAA], 2023). These two substances are associated with consequences such as driving under the influence, aggressive behavior (i.e., physical fights, property damage), missing schoolwork, and trouble sleeping (Bravo et al., 2019a, 2019b; Duckworth et al., 2024; Geisner et al., 2018; Jackson et al., 2020). Given the high rates of excessive alcohol and cannabis use and their related consequences on university campuses, research has focused on identifying protective factors that are associated with lower problematic substance use among this at-risk population.

One protective factor that has been identified is mindfulness. Mindfulness has been conceptualized as one’s ability to focus awareness on present-moment experiences in a non-judgmental and accepting way (Baer et al., 2006; Bishop et al., 2004; Bowlin & Baer, 2012; Roos et al., 2015). In recent decades, researchers and clinicians have linked trait mindfulness to both improved psychological and behavioral health outcomes, including lower substance use (Baer et al., 2006; Karyadi et al., 2014; Roos et al., 2015). Specific to university student drinking, prior research has found strong relationships between mindfulness, alcohol use behaviors, and alcohol-related consequences (Karyadi & Cyders, 2015; Ostafin et al., 2012; Wisener & Khoury, 2019). For example, a longitudinal study by Single et al. (2019) revealed that first-year university students high in trait mindfulness were less likely to engage in harmful alcohol use. Specifically, the mindfulness facets of acting with awareness (i.e., focusing awareness and full attention on one’s current activity or experiences), nonjudging of inner experience (i.e., experiencing thoughts and feelings without evaluating or criticizing oneself), and nonreactivity to inner experience (i.e., allowing thoughts and feelings to come and go without getting caught up in them) predicted decreased alcohol use 4 months later. However, describing (i.e., labeling experiences with words) and observing (i.e., noticing and attending to internal and external experiences) facets did not significantly predict alcohol use.

Associations between mindfulness, cannabis use, and cannabis-related consequences have also been established (Carlon et al., 2023; De Dios et al., 2012; Herchenroeder et al., 2022; Lin et al., 2021). For instance, Luba et al. (2020) discovered that increases in savoring, a form of mindfulness focused on positive affect, weakened the positive relationship between cannabis use and negative consequences, suggesting that mindfulness may play a role in reducing cannabis-related consequences above and beyond frequency of use. However, despite these findings, few studies have focused on examining possible mechanisms underlying these relationships. Indeed, researchers (e.g., Karyadi & Cyders, 2015; Wisener & Khoury, 2021) have stressed the importance of identifying variables that may influence the link between mindfulness and substance use. Thus, identifying intermediate variables that may account for the relationship between mindfulness and substance-related outcomes is warranted.

Motivational models of substance use provide a framework for how distinct motives influence decisions about substance use (Cooper et al., 2016; Korcha et al., 2011; Votaw & Witkiewitz, 2021). According to alcohol use models, four distinct alcohol use motives include coping (i.e., drinking to reduce or manage negative emotions), enhancement (i.e., drinking to increase enjoyable experiences or positive mood), social (i.e., drinking to celebrate and make social activities more enjoyable), and conformity motives (i.e., drinking due to peer and social pressure) (Cooper et al., 2016; Kuntsche et al., 2005). Each of these motives exhibit distinct associations with alcohol-related outcomes (Cooper et al., 2016). In addition to these four motives, research on cannabis use motives suggests that an additional factor, expansion of the mind (i.e., desire for new experiences that expand awareness), also plays a role in motivating individuals to engage in cannabis use (Bresin & Mekawi, 2019; Buckner, 2013; Simons et al., 1998).

There is theoretical support for why substance use motives may mediate the relationship between mindfulness and substance use outcomes. Focusing on coping motives as an example, coping motives are posited to be influential in repeated substance use as individuals may habitually use drugs to reduce negative affect (Bowen & Enkema, 2014; Khantzian, 2003). Hence, coping motives may function as a reinforcer in a maladaptive cycle of problematic substance use (Baker et al., 2004; Bowen & Enkema, 2014; Hides et al., 2008). Mindfulness involves focusing awareness on present-moment experiences in a non-judgmental and accepting way (Baer et al., 2006; Bishop et al., 2004; Bowlin & Baer, 2012) and is associated with engaging in more adaptive coping responses (Keng et al., 2018). This suggests that individuals high in trait mindfulness may be less likely to endorse substance use coping motivations when dealing with negative affect, which may reduce negative substance use outcomes (Bravo et al., 2016b).

Empirically, alcohol use motives have been shown to mediate the relationship between mindfulness and alcohol use outcomes (Clerkin et al., 2017; Leigh & Neighbors, 2009; Vinci et al., 2016). For example, Roos et al. (2015) found that mindfulness facets were negatively associated with alcohol use and problems via drinking motives among university students. Specifically, non-judging of inner experience, describing, and acting with awareness were related to lower alcohol use and problems via lower alcohol-coping motives. Additionally, describing and acting with awareness had indirect effects on alcohol problems via conformity motives and acting with awareness and nonjudging of inner experience had indirect effects on alcohol problems via enhancement motives. Bivariate links between mindfulness, cannabis use motives, and cannabis use outcomes have also been identified (Bonn-Miller et al., 2010; Karyadi et al., 2014; Wisener & Khoury, 2021). However, limited research has assessed the indirect effects of mindfulness on cannabis-related outcomes via cannabis use motives.

Though previous studies support indirect effects of mindfulness on substance use outcomes via substance use motives, previous studies have relied heavily on variable-centered statistical approaches (e.g., multiple regression, factor analysis, structural equation modeling). However, variable-centered approaches present two limitations: (1) they tend to only examine associations between a single mindfulness facet and related outcomes, and (2) they assume that samples represent a homogenous population. Within the mindfulness literature, several studies have used latent profile analyses (LPA; a person-centered approach that identifies distinct homogenous subgroups within a population based on continuous indicators, Collins & Lanza, 2009) to identify subpopulations of individuals on mindfulness facets (Bravo et al., 2016a, 2018; Pearson et al., 2015). Using LPA, Pearson et al. (2015) identified four groups of university students based on their scores on the Five Facet Mindfulness Questionnaire (FFMQ; Baer et al., 2006): Low Mindfulness (i.e., low-to-average scores on all mindfulness facets), High Mindfulness (i.e., moderately high scores on all mindfulness facets), Judgmentally Observing (i.e., high scores on observing, low scores on non-judging of inner experience and acting with awareness), and Non-Judgmentally Aware (i.e., low scores on observing, high scores on non-judging of inner experience and acting with awareness). Since this initial study, several other researchers have replicated this four-profile solution among university students and other populations (Bravo et al., 2016a, 2018; Kimmes et al., 2017).

Prior research has found strong relationships between mindfulness profiles and psychological health outcomes such as well-being, self-regulation, anxiety, and attachment (De Souza Marcovski & Miller, 2023; Gu et al., 2020; Kimmes et al., 2017; Lam et al., 2018). For example, in a sample of meditation-naïve and meditation-experienced university students, Bravo et al. (2016a) found that mindfulness profiles were associated with adaptive psychological and emotional outcomes. Specifically, the high mindfulness profile was associated with significantly higher psychological well-being, self-regulation, and psychological flexibility for both meditation-naïve and meditation-experienced university students. Further, both the high mindfulness and non-judgmentally aware profiles were associated with lower depressive symptoms, worry, rumination, and distress intolerance for both meditation-naïve and meditation-experienced university students. Studies focused on examining how mindfulness profiles differ on substance use outcomes are limited.

More recently, Carlon et al. (2023) used LPA to identify subtypes of dispositional mindfulness and their relationship with cannabis use behaviors and weekly alcohol use. The researchers discovered that the judgmentally observing profile had significantly more hazardous cannabis use and cannabis-related consequences compared with other profiles. However, they found no significant differences between mindfulness profiles and weekly alcohol use, which is inconsistent with previous studies that have found associations between mindfulness and problematic alcohol use in university students (e.g., Fernandez et al., 2010). It should be noted that the Carlon et al. (2023) study was restricted to cannabis users; thus, it may not reflect a representative sample of university student substance users.

Taken together, there is a dearth of research on whether mindfulness profiles differ on substance use outcomes (e.g., alcohol and cannabis), and few studies to date have investigated plausible mechanisms (i.e., alcohol and cannabis use motives) that could account for these relationships. Evaluating mindfulness profiles could potentially improve mindfulness-based interventions (MBIs) targeting substance use. Specifically, clinical work incorporating MBIs has a direct goal of cultivating mindfulness through a one-size-fit-all approach (i.e., using variable centered approaches), but this approach may not translate as well into clinical intervention work. For example, most MBIs are implemented on a specific clinical sample by typically providing the intervention as is to each participant (Bowen et al., 2014; Killeen et al., 2023; Tacón et al., 2004). However, this assumes that all patients and clients are coming in with the same level of trait mindfulness (i.e., low or high mindfulness). Prior work has shown that there are distinct mindfulness profiles across numerous populations such as cancer patients, adults (age 18 through 75 years), military personnel, and university students (Bravo et al., 2018; De Souza Marcovski & Miller, 2023; Lam et al., 2018), which may indicate a precision-medicine approach may be needed to improve the efficacy of MBIs by applying it to specific mindfulness profiles.

The purpose of the present study was to replicate and expand previous research on mindfulness, substance use motives, and substance use outcomes among university students. Specifically, we aimed to (1) identify distinct profiles of mindfulness among a sample of university students in the USA who use alcohol and cannabis, and (2) examine whether these mindfulness profiles are indirectly associated with alcohol and cannabis-related outcomes via alcohol and cannabis use motives. Based on prior research (Bravo et al., 2016a, 2018; Carlon et al., 2023; Karyadi & Cyders, 2015; Pearson et al., 2015), we expected that a distinct high mindfulness subgroup would emerge as the most salient profile associated with lower negative alcohol and cannabis use outcomes. Further, we hypothesized that the high mindfulness profile would be associated with lower negative alcohol and cannabis use–related consequences via lower scores on motives (particular coping) for alcohol and cannabis use compared to other profiles.

## Method

### Participants

The present study is a secondary analysis of a cross-national study focused on mental health, personality traits, and substance use behaviors (Bravo et al., 2021). Participants were university students recruited to participate in an online survey from seven countries (USA, Argentina, Spain, Uruguay, England, Canada, South Africa). Given that the present study aimed to directly replicate past research on mindfulness profiles among US university students, the analytic sample for this study was limited to 771 US students who completed the FFMQ (Baer et al., 2006) and endorsed using both alcohol and cannabis in the past 30 days. The majority of participants identified as White (75.7%) and female (66.8%), and reported a mean age of 19.50 (*Median* = 19.00; *SD* = 2.67) years.

### Procedure

Participants were recruited from Psychology Department research pools at five universities across four US states (Colorado, New Mexico, New York, Virginia) and received research participation credit applied to their courses for completing the survey. Specific to the present study, participants completed measures assessing trait mindfulness, alcohol and cannabis use motives, and alcohol and cannabis use and related consequences.

### Measures

Composite scores for each measure were created by either summing items, averaging items, or reverse-coding items when appropriate such that higher scores indicate higher levels of the construct. The bivariate correlations, descriptive statistics, and internal consistency measures in the present sample are shown in Table S1 of the Supplementary Information.

#### Trait Mindfulness

Trait mindfulness was assessed using the 39-item Five Facet Mindfulness Questionnaire (FFMQ; Baer et al., 2006) measured on a 5-point response scale (1 = *never or very rarely true*, 5 = *very often or always true*). The five facets (items were averaged) assessed by the FFMQ were as follows: Observing (*α* = 0.86), Describing (*α* = 0.79), Acting with Awareness (*α* = 0.90), Non-judging of Inner Experience (*α* = 0.92), Non-reactivity to Inner Experience (*α* = 0.83).

#### Alcohol Use

Alcohol use was assessed using several indicators: an indicator of past 30-day binge drinking frequency (i.e., past 30-day frequency of drinking 4 + /5 + standard drinks in a period of two hours or less for women/men; NIAAA, 2023), an indicator of typical frequency of alcohol use, and an indicator of typical quantity of alcohol use. Participants were first presented with a visual guide about typical drinks to help orient them to Standard Drink Units (SDUs). We assessed Typical Alcohol Frequency and Quantity using a grid such that each day of the week was broken down into six 4-hr blocks of time (12 a.m.–4 a.m., 4 a.m.–8 a.m., 8 a.m.–12 p.m., etc.) and participants were asked to report at which times they consumed alcohol during a “typical week” in the past 30 days, as well as the number of standard drinks consumed during that time block. Typical frequency of alcohol use was calculated by summing the total number of time blocks for which participants reported using alcohol during the typical week (ranges 0–42). Typical Quantity of alcohol use was calculated by summing the total number of SDUs consumed across time blocks during the typical week. Total number of SDUs consumed (summed) was transformed into grams of alcohol taking into account country-specific (in this case the USA) SDU rates based on grams of alcohol (quantity estimates > 3*SD*s above the mean were Winsorized).

#### Cannabis Use

Cannabis use was assessed using several indicators: an indicator of typical frequency of cannabis use and an indicator of typical quantity of cannabis use. Participants were presented with a visual guide showing different amounts of cannabis in grams. Typical Cannabis Use Frequency and Quantity were assessed using the Marijuana Use Grid (MUG; Pearson & Marijuana Outcomes Study Team, n.d.). Specifically, each day of the week was broken down into six 4-hr blocks of time (12 a.m.–4 a.m., 4 a.m.–8 a.m., 8 a.m.–12 p.m., etc.), and participants were asked to report at which times they used cannabis during a “typical week” in the past 30 days as well as the quantity of grams consumed during that time block. We calculated Typical Frequency of cannabis use by summing the total number of time blocks for which they reported using during the typical week (ranges 0–42). We calculated Typical Quantity of cannabis use by summing the total number of grams consumed across time blocks during the typical week (quantity estimates > 3*SD*s above the mean were Winsorized).

#### Alcohol Use Motives

Alcohol use motives were assessed using the Short-Form of the Drinking Motives Questionnaire-Revised (DMQ-R SF; Kuntsche & Kuntsche, 2009). Participants responded to the 12-item questionnaire using a 5-point scale (1 = *Almost never/never*, 5 = *Almost always/always*). Each item was a statement that concerned the frequency of drinking for four distinct drinking motives: Coping (3 items; e.g., because it helps me when I feel depressed or nervous; *α* = 0.83), Enhancement (3 items; e.g., because I like the feeling; *α* = 0.77), Social (3 items; e.g., because it makes social gatherings more fun; *α* = 0.90), Conformity (3 items; e.g., so I won’t feel left out; *α* = 0.87).

#### Cannabis Use Motives

Cannabis use motives were assessed using a short version of the Marijuana Motives Questionnaire (MMQ; Simons et al., 1998). Participants responded to the 15-item measure using a 5-point scale (1 = *Almost never/never*, 5 = *Almost always/always*). Each item was a statement that concerned the frequency of cannabis use for five distinct cannabis use motives: Coping (3 items; e.g., to forget my worries; *α* = 0.88), Enhancement (3 items; e.g., because I like the feeling; *α* = 0.86), Social (3 items; e.g., because it makes social gatherings more fun; *α* = 0.89), conformity (3 items; e.g., to fit in with the group I like; *α* = 0.90), and Expansion (3 items; e.g., because it helps me be more creative and original; *α* = 0.92).

#### Alcohol-Related and Cannabis-Related Problems

Alcohol use disorder (AUD) symptoms were assessed using the 10-item Alcohol Use Disorders Identification Test-US (USAUDIT; Higgins-Biddle & Babor, 2018; *α* = 0.80). Cannabis use disorder (CUD) symptoms were assessed using the 8-item Cannabis Use Disorder Identification Test-Revised (CUDIT-R; Adamson et al., 2010; *α* = 0.81). Past 30-day Alcohol-related Problems were assessed using the 24-item Brief-Young Adult Alcohol Consequences Questionnaire (BYAACQ; Kahler et al., 2005). Past 30-day Cannabis-related Problems were assessed using the 21-item Brief Marijuana Consequences Questionnaire (B-MACQ; Simons et al., 2012). For both the B-MACQ and BYAACQ, we summed all items to create a composite score reflective of the number of distinct alcohol (*α* = 0.86) and cannabis (*α* = 0.89) problems experienced in the past 30 days.

### Data Analyses

To determine the number of latent classes based on the pattern of means of the five subscales of the FFMQ across our analytic sample, we used the Lo-Mendell-Rubin adjusted likelihood ratio test (LRT, Lo et al., 2001; Vuong, 1989), which compares whether a *k* class solution fits better than a *k*–1 class solution using M*plus* 8.8 (Muthén & Muthén, 1998–2023). As recommended by previous research (Henson et al., 2007; Marsh et al., 2009; Nylund et al., 2007), we also examined goodness-of-fit indices (i.e., Akaike Information Criterion, Akaike, 1973, 1974; Bayesian Information Criterion, Schwarz, 1978), classification diagnostics (e.g., relative entropy [0.80 is considered high, Clark & Muthén, 2009] and mean posterior assignment probabilities [0.70 or higher is considered optimal, Nagin, 2005]), and substantive interpretation to settle upon the number of latent classes. Furthermore, Nagin (2005) suggests that when it is difficult to clearly identify an optimal number of groups (i.e., the LRT, goodness-of-fit indices and classification diagnostics offer different optimal class solutions), the most parsimonious model should be selected, and the smallest class of any class solution should not contain less than 5% of the sample.

After deciding on our class solutions, equality of means across the latent classes on distal outcomes was tested using the automatic BCH method (Asparouhov & Muthén, 2015; Bakk & Vermunt, 2016), which uses posterior probability-based multiple imputations (Asparouhov & Muthén, 2007). Outcomes included continuous scores on alcohol variables (i.e., Typical Alcohol Use Quantity and Frequency, past 30-day Binge Drinking Frequency, past 30-day Alcohol-related Problems, AUD symptoms, and alcohol use motives), as well as cannabis variables (i.e., Typical Cannabis Use Quantity and Frequency, past 30-day Cannabis-related Problems, CUD symptoms, and cannabis use motives).

#### Indirect Effect Analyses

To determine whether mindfulness profiles had indirect effects on alcohol-related and cannabis-related problems and AUD/CUD symptoms via alcohol and cannabis use motives, we conducted parallel mediation analyses (Model 4 in PROCESS) using PROCESS 3.4 macro for SPSS (Hayes, 2017). For all four indirect effect models, we followed the steps outlined by Hayes (2017) for using a multicategorical antecedent variable. Importantly, because we utilized a multicategorical antecedent variable, all effects are relative and reflect comparisons between the mindfulness profiles on outcomes. Gender and typical alcohol and cannabis consumption were included as covariates. Statistical significance was determined by 95% percentile-based bootstrap (10,000 samples) confidence intervals that do not contain zero.

## Results

Table 1 reports commonly used fit statistics for 1- through 6-class solutions for the analytic sample. Within our analytic sample (*n* = 771), the likelihood ratio test indicated that a 2-class solution fit better than a 1-class solution (*p* < 0.001), a 3-class solution fit better than a 2-class solution (*p* < 0.001), and a 4 class solution fit better than a 3-class solution (*p* < 0.001); however, a 5-class solution did not fit significantly better than a 4-class solution (*p* = 0.209). The AIC, BIC, and adjusted BIC decreased from a 1-class solution through a 6-class solution indicating an improved fit as the number of class solutions increased, suggesting that a 5-class solution may be optimal (Table 1). However, given the results of the Likelihood Ratio Test, and prior research (Bravo et al., 2016a; Pearson et al., 2015), we settled on the 4-class solution (Table 1).

**Table 1:** Fit statistics for 1- through 6-class solutions for Latent Profile Analysis (LPA) across samples

Fit Statistics	1	2	3	4	5	6
AIC	9043.67	8399.02	8139.27	**7930.99**	7826.13	7748.99
BIC	9090.15	8473.38	8241.52	**8061.12**	7984.15	7934.91
Adjusted BIC	9058.40	8422.58	8171.66	**7972.21**	7876.18	7807.89
Entropy	––-	0.923	0.825	**0.818**	0.833	0.831
Smallest *n*	771	100	91	**92**	53	34
LRT	––-	*p* < 0.001	*p* < 0.001	***p* < 0.001**	*p* = 0.209	*p* = 0.370

*AIC* Akaike information criterion, *BIC* Bayesian information criterion

**Fig. 1:**
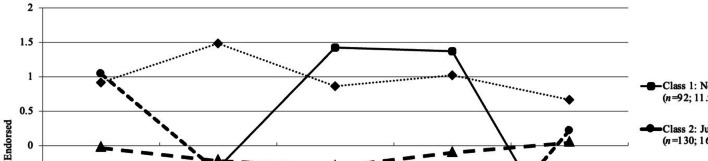
Depiction of the four latent classes defined by pattern of standardized means on five facets of mindfulness among university students (*n* = 771)

Figure 1 depicts the pattern of means (standardized) across the 4-latent classes. Class 1 comprised 11.93% of the sample (*n* = 92). This class was labeled the non-judgmentally aware group because participants in that group were high on Non-judging of Inner Experience (*z* = 1.38) and Acting with Awareness (*z* = 1.42), but very low on the Observing facet of mindfulness (*z* =  − 1.71). Class 2 comprised 16.86% of the sample (*n* = 130). This class was labeled the judgmentally observing group because participants in that group were the highest on Observing (*z* = 1.04), but very low on Non-judging of Inner Experience (*z* =  − 1.76) and Acting with Awareness (*z* =  − 1.42). Class 3 was the largest group and comprised 58.37% of the sample (*n* = 450). This class was labeled the low mindfulness group because participants in that group were relatively low on every facet of mindfulness (− 0.30 < *z* < 0.04). Finally, class 4 comprised 12.84% of the sample (*n* = 99). This class was labeled the high mindfulness group because participants in that group were moderately high on all facets of mindfulness (0.67 < *z* < 1.49).

### Mean Differences on Substance Use Outcomes

Across alcohol outcomes (Table 2), we found that the high mindfulness group generally had the most adaptive alcohol outcomes (i.e., lower Binge Drinking Frequency, Alcohol-related Problems, AUD symptoms, and Coping motivation). In contrast, the judgmentally observing group generally had the most maladaptive alcohol outcomes (i.e., greater Alcohol-related Problems, AUD symptoms, and Coping and Conformity motivations). The low mindfulness group had significantly higher AUD symptoms, Coping and Conformity motivations, and Alcohol-related problems compared to the high mindfulness group. In addition, we found that the non-judgmentally aware group had significantly lower Coping motivations and Alcohol-related Problems compared to the judgmentally observing and low mindfulness groups.

**Table 2:** Mean comparisons between latent classes on mindfulness facets and alcohol and cannabis outcomes among university students

	Class 1: Non-Judgmentally Aware	Class 2: Judgmentally Observing	Class 3: Low Mindfulness	Class 4: High Mindfulness	
*Mindfulness facets*	*M* (*SE*)	*M* (*SE*)	*M* (*SE*)	*M* (*SE*)	Class significant differences
Observing	−1.71 (0.068)	1.04 (0.084)	−0.03 (0.035)	0.92 (0.092)	2,4 > 3 > 1
Describing	−0.32 (0.082)	−0.38 (0.115)	−0.23 (0.043)	1.49 (0.102)	4 > 1,2,3
Acting with Awareness	1.42 (0.058)	−1.42 (0.083)	−0.31 (0.034)	0.86 (0.091)	1 > 4 > 3 > 2
Non-Judging	1.38 (0.052)	−1.76 (0.060)	−0.11 (0.032)	1.02 (0.069)	1 > 4 > 3 > 2
Non-Reactivity	−1.55 (0.084)	0.22 (0.109)	0.04 (0.036)	0.67 (0.103)	4 > 2,3 > 1
*Alcohol outcomes*	*M* (*SE*)	*M* (*SE*)	*M* (*SE*)	*M* (*SE*)	Class significant differences
Typical Quantity in Grams	181.68 (16.955)	165.86 (14.733)	158.50 (6.963)	136.03 (13.610)	1 > 4
Typical Frequency	3.86 (0.362)	4.10 (0.383)	3.79 (0.163)	3.70 (0.384)	None
Binge Drinking Frequency	3.17 (0.410)	3.13 (0.345)	3.17 (0.200)	2.09 (0.299)	1,2,3 > 4
Past 30-day Problems	4.85 (0.399)	7.21 (0.506)	5.89 (0.251)	3.50 (0.416)	2 > 3 > 1 > 4
AUD Symptoms	9.83 (0.614)	12.18 (0.744)	10.72 (0.308)	8.65 (0.567)	2 > 1,4; 3 > 4
Social Motives	3.40 (0.116)	3.70 (0.119)	3.43 (0.060)	3.38 (0.144)	None
Coping Motives	1.74 (0.091)	2.57 (0.122)	1.98 (0.051)	1.41 (0.085)	2 > 3 > 1 > 4
Enhancement Motives	3.02 (0.118)	3.59 (0.112)	3.14 (0.055)	3.52 (0.129)	2,4 > 1,3
Conformity Motives	1.50 (0.080)	1.95 (0.118)	1.68 (0.048)	1.26 (0.075)	2 > 1,3 > 4
*Cannabis outcomes*	*M* (*SE*)	*M* (*SE*)	*M* (*SE*)	*M* (*SE*)	Class significant differences
Typical Quantity in Grams	5.78 (0.928)	6.83 (0.905)	5.45 (0.492)	5.85 (0.167)	None
Typical Frequency	5.78 (0.879)	7.88 (0.922)	6.30 (0.452)	6.97 (0.983)	None
Past 30-day Problems	1.60 (0.219)	5.90 (0.565)	3.62 (0.221)	2.10 (0.400)	2 > 3 > 1,4
CUD Symptoms	6.27 (0.558)	10.67 (0.755)	8.17 (0.298)	6.50 (0.600)	2 > 3 > 1,4
Social Motives	2.26 (0.137)	2.75 (0.138)	2.30 (0.063)	2.24 (0.155)	2 > 1,3,4
Coping Motives	2.05 (0.124)	3.08 (0.143)	2.23 (0.066)	1.77 (0.126)	2 > 1,3,4; 3 > 4
Enhancement Motives	3.50 (0.134)	4.06 (0.112)	3.50 (0.062)	4.02 (0.144)	2,4 > 1,3
Conformity Motives	1.20 (0.062)	1.65 (0.106)	1.39 (0.038)	1.12 (0.053)	2 > 3 > 1,4
Expansion Motives	1.92 (0.128)	2.82 (0.150)	2.18 (0.066)	2.38 (0.166)	2 > 4 > 1; 2 > 3

Mindfulness scores are presented as standardized values. Negative mindfulness scores indicate below the total sample mean and positive mindfulness scores indicate above the total sample mean. *AUD* alcohol use disorder, *CUD* cannabis use disorder

On cannabis outcomes (Table 2), we found that the high mindfulness and non-judgmentally aware groups generally had the most adaptive cannabis outcomes (i.e., lower Cannabis-related problems, CUD symptoms, and Social, Coping, Conformity, and Expansion motivations) and did not differ from each other except on Expansion motives (higher for the high mindfulness group). In contrast, the judgmentally observing group had the most maladaptive cannabis outcomes (i.e., higher Cannabis-related Problems, CUD symptoms, and Social, Coping, Conformity, and Expansion motivations). The low mindfulness group typically endorsed greater cannabis outcomes than the high mindfulness and non-judgmentally aware groups.

### Indirect Effect Models

Participants who had less than a 75% probability on their most likely class were excluded from the mediation analyses in order to reduce potential bias in comparisons across profiles. Nonetheless, analyses were conducted among the full analytic sample (Tables S2 and S3) and results were largely consistent with our more conservative analytic sample (i.e., only among individuals with 75% or greater probability for a specific profile). For each analysis, the low mindfulness profile was used as the reference group.

#### Low Mindfulness vs. Judgmentally Observing

As shown in Table 3, the judgmentally observing group reported greater endorsement of Social and Coping motivations to drink compared to the low mindfulness group, which in turn were associated with greater past 30-day alcohol-related problems. Further, the judgmentally observing group reported greater endorsement of Coping, Enhancement, and Conformity motivations to drink compared to the low mindfulness group, which in turn were associated with greater AUD symptoms.

**Table 3:** Summary of relative total, relative indirect, and relative direct effects of alcohol mediation model with low mindfulness profile as the reference group (*n* = 591)

Outcome variable: **Past 30-day Alcohol Problems**	*B*	95% CI
Relative direct effects		
X1 > Alcohol Problems	0.51	− 0.43, 1.44
Relative indirect effects		
X1 > Social Motives > Alcohol Problems	**0.16**	**0.01, 0.36**
X1 > Coping Motives > Alcohol Problems	**0.40**	**0.14, 0.74**
X1 > Enhancement Motives > Alcohol Problems	0.03	− 0.16, 0.25
X1 > Conformity Motives > Alcohol Problems	0.16	− 0.001, 0.43
Relative total effect		
Relative Direct + Relative Indirect	**1.26**	**0.31, 2.21**
Outcome variable: **Past 30-day Alcohol Problems**	*B*	95% CI
Relative direct effects		
X2 > Alcohol Problems	** − 1.19**	** − 2.15, − 0.23**
Relative indirect effects		
X2 > Social Motives > Alcohol Problems	− 0.03	− 0.16, 0.10
X2 > Coping Motives > Alcohol Problems	** − 0.16**	** − 0.36, − 0.01**
X2 > Enhancement Motives > Alcohol Problems	− 0.02	− 0.14, 0.09
X2 > Conformity Motives > Alcohol Problems	− 0.10	− 0.26, 0.01
Relative total effect		
Relative Direct + Relative Indirect	**− 1.49**	**− 2.49, − 0.49**
Outcome variable: **Past 30-day Alcohol Problems**	*B*	95% CI
Relative direct effects		
X3 > Alcohol Problems	** − 1.52**	** − 2.53, − 0.50**
Relative indirect effects		
X3 > Social Motives > Alcohol Problems	− 0.07	− 0.25, 0.08
X3 > Coping Motives > Alcohol Problems	** − 0.37**	** − 0.62, − 0.15**
X3 > Enhancement Motives > Alcohol Problems	0.01	− 0.08, 0.14
X3 > Conformity Motives > Alcohol Problems	** − 0.22**	** − 0.45, − 0.04**
Relative total effect		
Relative Direct + Relative Indirect	** − 2.16**	** − 3.20, − 1.13**
Outcome variable: **Alcohol Use Disorder Symptoms**	*B*	95% CI
Relative direct effects		
X1 > AUD Symptoms	0.65	− 0.40, 1.69
Relative indirect effects		
X1 > Social Motives > AUD Symptoms	0.13	− 0.01, 0.34
X1 > Coping Motives > AUD Symptoms	**0.53**	**0.21, 0.96**
X1 > Enhancement Motives > AUD Symptoms	**0.32**	**0.08, 0.64**
X1 > Conformity Motives > AUD Symptoms	**0.22**	**0.01, 0.58**
Relative total effects		
Relative Direct + Relative Indirect	**1.84**	**0.73, 2.96**
: **Alcohol Use Disorder Symptoms**	*B*	95% CI
Relative direct effects		
X2 > AUD Symptoms	− 0.99	− 2.07, 0.09
Relative indirect effects		
X2 > Social Motives > AUD Symptoms	− 0.02	− 0.15, 0.09
X2 > Coping Motives > AUD Symptoms	** − 0.21**	** − 0.46, − 0.01**
X2 > Enhancement Motives > AUD Symptoms	− 0.17	− 0.42, 0.01
X2 > Conformity Motives > AUD Symptoms	− 0.13	− 0.33, 0.01
Relative total effect		
Relative Direct + Relative Indirect	** − 1.53**	** − 2.69, − 0.36**
Outcome variable: **Alcohol Use Disorder Symptoms**	*B*	95% CI
Relative direct effects		
X3 > AUD Symptoms	− 0.59	− 1.73, 0.56
Relative indirect effects		
X3 > Social Motives > AUD Symptoms	− 0.06	− 0.24, 0.07
X3 > Coping Motives > AUD Symptoms	** − 0.49**	** − 0.83, − 0.22**
X3 > Enhancement Motives > AUD Symptoms	0.14	− 0.04, 0.40
X3 > Conformity Motives > AUD Symptoms	** − 0.29**	** − 0.59, − 0.07**
Relative total effect		
Relative Direct + Relative Indirect	** − 1.28**	** − 2.49, − 0.07**

Significant effects are in bold typeface for emphasis and were determined by a 95% percentile bootstrap confidence interval (based on 10,000 bootstrap samples) that does not contain zero. For clarity, two models were conducted with past 30-day alcohol problems and AUD symptoms estimated separately as outcomes. However, for parsimony, results are presented by the profile comparison effects for each outcome. For all models, X1 = Low Mindfulness profile compared to Judgmentally Observing profile, X2 = Low Mindfulness profile compared to Non-Judgmentally Aware profile, and X3 = Low Mindfulness profile compared to High Mindfulness profile. Effects from covariates (i.e., gender and typical alcohol consumption) were not included for parsimony but are available at the OSF website

As shown in Table 4, the judgmentally observing group reported greater endorsement of Coping and Enhancement motivations compared to the low mindfulness group, which in turn were associated with greater past 30-day Cannabis-related Problems. Further, the judgmentally observing group reported greater endorsement of Coping, Enhancement, and Expansion motivations compared to the low mindfulness profile, which in turn were associated with greater CUD symptoms. Conversely, the judgmentally observing group reported lower endorsement of Social motivation to use cannabis compared to the low mindfulness profile, which in turn was associated with lower CUD symptoms.

**Table 4:** Summary of relative total, relative indirect, and relative direct effects of cannabis mediation model with low mindfulness profile as the reference group (*n* = 561)

Outcome variable: **Past 30-day Cannabis Problems**	*B*	95% CI
Relative direct effects		
X1 > Cannabis Problems	**1.16**	**0.23, 2.09**
Relative indirect effects		
X1 > Social Motives > Cannabis Problems	− 0.12	− 0.32, 0.03
X1 > Coping Motives > Cannabis Problems	**0.80**	**0.43, 1.23**
X1 > Enhancement Motives > Cannabis Problems	**0.27**	**0.10, 0.49**
X1 > Conformity Motives > Cannabis Problems	0.15	− 0.01, 0.42
X1 > Expansion Motives > Cannabis Problems	0.04	− 0.17, 0.28
Relative total effect		
Relative Direct + Relative Indirect	**2.31**	**1.34, 3.27**
**Past 30-day Cannabis Problems**	*B*	95% CI
Relative direct effects		
X2 > Cannabis Problems	** − 1.99**	** − 2.92, − 1.06**
Relative indirect effects		
X2 > Social Motives > Cannabis Problems	− 0.004	− 0.10, 0.09
X2 > Coping Motives > Cannabis Problems	− 0.10	− 0.38, 0.15
X2 > Enhancement Motives > Cannabis Problems	0.01	− 0.14, 0.16
X2 > Conformity Motives > Cannabis Problems	− 0.09	− 0.28, 0.01
X2 > Expansion Motives > Cannabis Problems	− 0.01	− 0.11, 0.05
Relative total effect		
Relative Direct + Relative Indirect	** − 2.19**	** − 3.18, − 1.20**
Outcome variable: **Past 30-day Cannabis Problems**	*B*	95% CI
Relative direct effects		
X3 > Cannabis Problems	** − 1.35**	** − 2.33, − 0.38**
Relative indirect effects		
X3 > Social Motives > Cannabis Problems	0.04	− 0.04, 0.17
X3 > Coping Motives > Cannabis Problems	** − 0.40**	** − 0.71, − 0.13**
X3 > Enhancement Motives > Cannabis Problems	**0.18**	**0.03, 0.39**
X3 > Conformity Motives > Cannabis Problems	− 0.15	− 0.33, 0.00
X3 > Expansion Motives > Cannabis Problems	0.004	− 0.06, 0.08
Relative total effect		
Relative Direct + Relative Indirect	** − 1.67**	** − 2.69, − 0.66**
Outcome variable: **Cannabis Use Disorder Symptoms**	*B*	95% CI
Relative direct effects		
X1 > CUD Symptoms	0.79	− 0.35, 1.93
Relative indirect effects		
X1 > Social Motives > CUD Symptoms	** − 0.21**	** − 0.50, − 0.01**
X1 > Coping Motives > CUD Symptoms	**1.09**	**0.60, 1.63**
X1 > Enhancement Motives > CUD Symptoms	**0.41**	**0.16, 0.71**
X1 > Conformity Motives > CUD Symptoms	0.18	− 0.02, 0.48
X1 > Expansion Motives > CUD Symptoms	**0.35**	**0.08, 0.72**
Relative total effects		
Relative Direct + Relative Indirect	**2.60**	**1.37, 3.82**
Outcome variable: **Cannabis Use Disorder Symptoms**	*B*	95% CI
Relative direct effects		
X2 > CUD Symptoms	** − 1.60**	** − 2.73, − 0.46**
Relative indirect effects		
X2 > Social Motives > CUD Symptoms	− 0.01	− 0.18, 0.14
X2 > Coping Motives > CUD Symptoms	− 0.14	− 0.51, 0.20
X2 > Enhancement Motives > CUD Symptoms	0.01	− 0.22, 0.23
X2 > Conformity Motives > CUD Symptoms	− 0.11	− 0.25, 0.03
X2 > Expansion Motives > CUD Symptoms	− 0.09	− 0.29, 0.07
Relative total effect		
Relative Direct + Relative Indirect	** − 1.93**	** − 3.19, − 0.67**
Outcome variable: **Cannabis Use Disorder Symptoms**	*B*	95% CI
Relative direct effects		
X3 > CUD Symptoms	− 1.10	− 2.29, 0.10
Relative indirect effects		
X3 > Social Motives > CUD Symptoms	0.07	− 0.06, 0.26
X3 > Coping Motives > CUD Symptoms	** − 0.54**	** − 0.95, − 0.17**
X3 > Enhancement Motives > CUD Symptoms	**0.27**	**0.04, 0.57**
X3 > Conformity Motives > CUD Symptoms	− 0.18	− 0.37, 0.01
X3 > Expansion Motives > CUD Symptoms	0.04	− 0.17, 0.25
Relative total effect		
Relative Direct + Relative Indirect	** − 1.43**	** − 2.72, − 0.14**

Significant effects are in bold typeface for emphasis and were determined by a 95% percentile bootstrap confidence interval (based on 10,000 bootstrap samples) that does not contain zero. For clarity, two models were conducted with past 30-day cannabis problems and CUD symptoms estimated separately as outcomes. However, for parsimony, results are presented by the profile comparison effects for each outcome. For all models, X1 = Low Mindfulness profile compared to Judgmentally Observing profile, X2 = Low Mindfulness profile compared to Non-Judgmentally Aware profile, and X3 = Low Mindfulness profile compared to High Mindfulness profile. Effects from covariates (i.e., gender and typical cannabis consumption) were not included for parsimony but are available at the OSF website

#### Low Mindfulness vs. Non-Judgmentally Aware

As shown in Table 3, the non-judgmentally aware group reported lower endorsement of Coping motivation to drink compared to the low mindfulness profile, which in turn was associated with lower past 30-day Alcohol-related Problems and AUD symptoms. As shown in Table 4, there were no significant indirect effects of the non-judgmentally aware group on cannabis outcomes via cannabis use motives.

#### Low Mindfulness vs. High Mindfulness

As shown in Table 3, the high mindfulness group reported lower endorsement of Coping and Conformity motivations to drink compared to the low mindfulness profile, which in turn were associated with lower past 30-day alcohol-related problems and AUD symptoms. As shown in Table 4, the high mindfulness group reported lower endorsement of Coping motivation compared to the low mindfulness profile, which in turn was associated with lower past 30-day cannabis-related problems and CUD symptoms. Conversely, being in the high mindfulness group was associated with greater endorsement of Enhancement motivation to use cannabis compared to the low mindfulness profile, which in turn was associated with greater past 30-day cannabis-related problems and CUD symptoms.

## Discussion

The present study sought to replicate and expand previous research on mindfulness, substance use motives, and substance use outcomes among university students by identifying distinct profiles of mindfulness among a sample of university students in the USA who consumed alcohol and cannabis in the past month. Further, we examined whether mindfulness profiles had indirect effects on alcohol and cannabis outcomes via alcohol and cannabis use motives. In line with previous LPA mindfulness research (Bravo et al., 2016a, 2018; Kimmes et al., 2017; Pearson et al., 2015), we identified four classes of individuals based on their mindfulness scores that included a high mindfulness group (i.e., moderately high scores on all mindfulness facets), a low mindfulness group (i.e., low-to-average scores on all mindfulness facets), a judgmentally observing group (i.e., high scores on Observing, low scores on Non-judging of Inner Experience and Acting with Awareness facets), and non-judgmentally aware group (i.e., low scores on Observing, high scores on Non-judging of Inner Experience and Acting with Awareness facets).

The mindfulness profiles identified differed significantly on alcohol and cannabis use outcomes. Specifically, we found that the high mindfulness group generally had the most adaptive alcohol outcomes (i.e., lower Binge Drinking Frequency, Alcohol-related Problems, AUD symptoms, and Coping motivation) while the judgmentally observing group generally had the most maladaptive alcohol outcomes (i.e., greater Alcohol-related Problems, AUD symptoms, and Coping and Conformity motivations). On cannabis outcomes, we found that the high mindfulness and non-judgmentally aware groups generally had the most adaptive cannabis outcomes (i.e., lower Cannabis-related Problems, CUD symptoms, and Social, Coping, Conformity, and Expansion motivations), whereas the judgmentally observing group had the most maladaptive cannabis outcomes (i.e., higher Cannabis-related Problems, CUD symptoms, and Social, Coping, Conformity, and Expansion motivations). These findings are consistent with existing mindfulness profile literature, which found that the high mindfulness group had the most adaptive mental health outcomes, such as higher psychological well-being, self-regulation, and psychological flexibility, whereas the judgmentally observing group had the most maladaptive mental health outcomes, such as higher depressive symptoms, worry, rumination, and distress intolerance (Bravo et al., 2016a, 2018; De Souza Marcovski & Miller, 2023; Gu et al., 2020; Lam et al., 2018).

Within our indirect effect models, there was evidence of indirect effects of mindfulness profiles on Alcohol and Cannabis-related Problems via alcohol and cannabis use motives. As expected, the high mindfulness group was less likely to endorse Coping and Conformity motivations to drink, which in turn were associated with lower past 30-day Alcohol-related Problems and AUD symptoms (compared to the low mindfulness group). In contrast, the judgmentally observing group was more likely to endorse Social and Coping motivations to drink, which in turn were associated with greater past 30-day Alcohol-related Problems (compared to the low mindfulness group). On AUD symptoms, the judgmentally observing group was more likely to endorse Coping, Enhancement, and Conformity motivations to drink, which in turn were associated with greater AUD symptoms (compared to the low mindfulness group). These results are consistent with prior research that has found associations between higher levels of trait mindfulness and lower alcohol-related problems via lower coping, enhancement, and conformity motives to drink (Roos et al., 2015; Vinci et al., 2016).

In our cannabis indirect effect models, the high mindfulness group was less likely to endorse Coping motivation to use cannabis, which in turn was associated with lower past 30-day Cannabis-related Problems and CUD symptoms (compared to the low mindfulness group). Conversely, the judgmentally observing group was more likely to endorse Coping and Enhancement motivations to use cannabis, which in turn were associated with greater past 30-day Cannabis-related Problems (compared to the low mindfulness group). Focusing on CUD symptoms, the judgmentally observing group was more likely to endorse Coping, Enhancement, and Expansion motivations, which in turn were associated with greater CUD symptoms (compared to the low mindfulness group). These findings align with previous studies that have identified links between mindfulness, cannabis use motives, and cannabis use outcomes (Bonn-Miller et al., 2010; Karyadi et al., 2014; Wisener & Khoury, 2021).

### Limitations and Future Research

The present study has several limitations that should be considered. First, our cross-sectional study design prevents the examination of true mediation in that we are unable to establish temporal precedence or make causal inferences. However, our findings of the indirect effects of mindfulness profiles on substance-related outcomes support the plausibility that substance use motives influence these associations. Future longitudinal LPA research (i.e., longitudinal LPA and latent transition analysis) as well as experimental studies should examine these relationships to test true mediation. Second, this study utilized the Five Facet Mindfulness Questionnaire (FFMQ; Baer et al., 2006) to measure trait mindfulness. Though the FFMQ is a reliable and valid measure of trait mindfulness (Christopher et al., 2012; Shallcross et al., 2020), some researchers have argued that the observing subscale requires refinement to improve construct validity (Rudkin et al., 2018). Moreover, there are many other trait mindfulness measures that exist (see Medvedev et al., 2022), depending on one’s conceptualization of trait mindfulness (Bravo et al., 2022). Thus, more research employing these other existing measures of trait mindfulness and how these measures may play a role in mindfulness profiles is needed. Third, despite the recruitment of a large multi-site sample of university students, the sample was relatively homogenous in terms of race and gender. Fourth, our sample was restricted to university students from the USA and future research needs to replicate this work across other samples (e.g., non-university attending young adults, clinical populations).

Despite limitations, this study has implications that should be considered. As previous studies have noted, the university environment increases vulnerability to engaging in excessive alcohol and cannabis use (Primack et al., 2012; Stone et al., 2012; Suerken et al., 2014). Thus, it is important to identify and target protective factors associated with lower problematic alcohol and cannabis use on university campuses. Mindfulness is one protective factor that has been identified, and studies on potential treatments have used mindfulness-based interventions to target and reduce problematic alcohol and cannabis use in university students (Mermelstein & Garske, 2015; Soriano-Ayala et al., 2020). In our study, we identified four mindfulness profiles and found that students in the high mindfulness group generally had the most adaptive alcohol and cannabis outcomes while the judgmentally observing group generally had the most maladaptive alcohol and cannabis outcomes. Therefore, mindfulness-based interventions targeting problematic alcohol and cannabis use in university students should consider mindfulness profiles to improve the efficacy of such interventions. These interventions may aim to teach more non-judging of inner experience and acting with awareness mindfulness skills, particularly for those presenting similar scores as those in the judgmentally observing profile.

Because mindfulness profiles were associated with alcohol and cannabis use motives and in turn alcohol and cannabis-related problems and AUD/CUD symptoms, two additional implications should be considered. First, motivational models of substance use and research on trait mindfulness theorize that distinct motives such as coping influences decisions about substance use (Cooper et al., 2016; Korcha et al., 2011; Votaw & Witkiewitz, 2021), and that higher trait mindfulness may relate to lower coping motives and subsequently lower substance use-related outcomes (Bowen & Enkema, 2014; Vinci et al., 2016). Our results provide support for motivational frameworks of substance use through the lens of mindfulness profiles and suggests that these mindfulness profiles may provide new pathways for understanding how mindfulness works as a protective factor. Second, the indirect effects of alcohol and cannabis use motives suggest that alcohol and cannabis use motives should also be targeted in mindfulness-based interventions for problematic alcohol and cannabis use among university students. For example, mindfulness-based interventions targeting substance use on university campuses could emphasize challenging alcohol and cannabis coping motives by teaching adaptive coping management skills.

In conclusion**,** the present study identified four distinct subgroups of individuals based on their mindfulness profiles in line with previous studies (Bravo et al., 2016a, 2018; Kimmes et al., 2017; Pearson et al., 2015) among a sample of university students in the USA who consumed alcohol and cannabis in the past month. Our findings suggest that individuals in the high mindfulness group (i.e., moderately high scores on all mindfulness facets) had the most adaptive alcohol and cannabis outcomes while the judgmentally observing group (i.e., high scores on Observing, low scores on Non-judging of Inner Experience and Acting with Awareness facets) generally had the most maladaptive alcohol and cannabis outcomes. Further, we found that the high mindfulness group was associated with lower endorsement of alcohol and cannabis Coping motives, which in turn was associated with lower Alcohol and Cannabis-related Problems and AUD/CUD symptoms (compared to the low mindfulness profile). Comparably, the judgmentally observing group was associated with higher endorsement of alcohol and cannabis Coping motives, which in turn was associated with greater Alcohol and Cannabis-related Problems and AUD/CUD symptoms (compared to the low mindfulness profile). These findings suggest that mindfulness-based interventions for problematic alcohol and cannabis use in university students should consider mindfulness profiles and improve efficacy through increasing non-judging of inner experience and acting with awareness mindfulness skills, while also focusing on alcohol and cannabis use motivations (particularly coping motivations).

## Supplementary Information

Below is the link to the electronic supplementary material.

Supplementary file1 (DOCX 42 KB)

## Data Availability

Data and analytic outputs are available at 10.17605/OSF.IO/8SQ5V.

## Declarations

### Use of Artificial Intelligence

Artificial intelligence was not used in this study.

### Disclaimer

NIAAA had no role in the study design, collection, analysis or interpretation of the data, writing the manuscript, or the decision to submit the paper for publication.
