# Supplementary material for: Mindfulness Profiles and Substance Use Outcomes in University Students: The Role of Alcohol and Cannabis Use Motives
Source: Mindfulness (N Y). 2025 Feb 28;16(4):1046–60. doi: 10.1007/s12671-025-02544-5 (PMC11993505; doi:10.1007/s12671-025-02544-5)
Supplement: Supplementary file 1 — Supplementary file1 (DOCX 42 KB) [file 12671_2025_2544_MOESM1_ESM.docx]

Table S1

*Bivariate correlations among study variables*

|  | 1 | 2 | 3 | 4 | 5 | 6 | 7 | 8 | 9 | 10 | 11 | 12 | 13 | 14 | 15 | 16 | 17 | 18 | 19 | 20 | 21 | 22 | 23 | *M* | *SD* |
| --- | --- | --- | --- | --- | --- | --- | --- | --- | --- | --- | --- | --- | --- | --- | --- | --- | --- | --- | --- | --- | --- | --- | --- | --- | --- |
| 1. Observing | 0.86 |  |  |  |  |  |  |  |  |  |  |  |  |  |  |  |  |  |  |  |  |  |  | 3.05 | 0.82 |
| 2. Describing | **0.25** | 0.79 |  |  |  |  |  |  |  |  |  |  |  |  |  |  |  |  |  |  |  |  |  | 3.15 | 0.67 |
| 3. Acting with Awareness | **-0.40** | **0.20** | 0.90 |  |  |  |  |  |  |  |  |  |  |  |  |  |  |  |  |  |  |  |  | 3.23 | 0.83 |
| 4. Non-Judging | **-0.46** | **0.16** | **0.67** | 0.92 |  |  |  |  |  |  |  |  |  |  |  |  |  |  |  |  |  |  |  | 3.29 | 0.90 |
| 5. Non-Reactivity | **0.58** | **0.32** | **-0.30** | **-0.27** | 0.83 |  |  |  |  |  |  |  |  |  |  |  |  |  |  |  |  |  |  | 2.80 | 0.70 |
| 6. ALC Typical Quantity | -0.06 | -0.08 | -0.03 | -0.03 | -0.04 | --- |  |  |  |  |  |  |  |  |  |  |  |  |  |  |  |  |  | 159.36 | 128.78 |
| 7. ALC Typical Frequency | 0.01 | -0.07 | -0.05 | -0.06 | -0.01 | **0.77** | --- |  |  |  |  |  |  |  |  |  |  |  |  |  |  |  |  | 3.84 | 3.09 |
| 8. ALC Problems | 0.05 | **-0.14** | **-0.23** | **-0.18** | 0.03 | **0.42** | **0.35** | 0.86 |  |  |  |  |  |  |  |  |  |  |  |  |  |  |  | 5.65 | 4.57 |
| 9. Binge Drinking Frequency | -0.03 | **-0.10** | -0.08 | -0.03 | -0.02 | **0.66** | **0.49** | **0.43** | --- |  |  |  |  |  |  |  |  |  |  |  |  |  |  | 3.01 | 3.56 |
| 10. AUD symptoms | 0.03 | **-0.11** | **-0.19** | **-0.13** | 0.04 | **0.60** | **0.46** | **0.62** | **0.64** | 0.80 |  |  |  |  |  |  |  |  |  |  |  |  |  | 10.57 | 5.96 |
| 11. ALC Social Motives | 0.01 | -0.01 | **-0.11** | -0.05 | 0.04 | **0.26** | **0.12** | **0.32** | **0.28** | **0.37** | 0.90 |  |  |  |  |  |  |  |  |  |  |  |  | 3.47 | 1.14 |
| 12. ALC Coping Motives | 0.09 | **-0.19** | **-0.23** | **-0.29** | -0.01 | **0.18** | **0.16** | **0.34** | **0.21** | **0.34** | **0.24** | 0.83 |  |  |  |  |  |  |  |  |  |  |  | 1.97 | 0.99 |
| 13. ALC Enhancement Motives | 0.09 | 0.04 | **-0.13** | -0.08 | 0.09 | **0.32** | **0.23** | **0.30** | **0.33** | **0.42** | **0.66** | **0.31** | 0.77 |  |  |  |  |  |  |  |  |  |  | 3.25 | 1.06 |
| 14. ALC Conformity Motives | 0.05 | **-0.14** | **-0.18** | **-0.15** | 0.06 | 0.03 | 0.01 | **0.26** | 0.10 | **0.24** | **0.36** | **0.35** | **0.24** | 0.87 |  |  |  |  |  |  |  |  |  | 1.64 | 0.91 |
| 15. CAN Typical Quantity | 0.05 | -0.00 | 0.00 | -0.07 | -0.01 | **0.18** | **0.21** | 0.05 | 0.13 | 0.03 | -0.05 | 0.02 | 0.01 | -0.02 | --- |  |  |  |  |  |  |  |  | 5.78 | 8.57 |
| 16. CAN Typical Frequency | **0.11** | 0.01 | 0.00 | -0.07 | 0.02 | **0.20** | **0.29** | 0.06 | 0.10 | 0.07 | 0.00 | 0.01 | 0.06 | 0.03 | **0.69** | --- |  |  |  |  |  |  |  | 6.59 | 7.94 |
| 17. CAN Problems | **0.20** | -0.05 | **-0.24** | **-0.27** | **0.12** | 0.09 | **0.10** | **0.33** | 0.06 | **0.17** | **0.13** | **0.16** | **0.14** | **0.18** | **0.26** | **0.35** | 0.89 |  |  |  |  |  |  | 3.54 | 4.26 |
| 18. CUD Symptoms | **0.16** | -0.02 | **-0.15** | **-0.20** | 0.08 | **0.11** | **0.11** | **0.22** | 0.07 | **0.17** | **0.12** | **0.13** | **0.13** | **0.16** | **0.43** | **0.55** | **0.76** | 0.81 |  |  |  |  |  | 8.13 | 5.90 |
| 19. CAN Social Motives | 0.05 | 0.01 | -0.08 | **-0.10** | 0.07 | 0.03 | 0.04 | **0.10** | -0.02 | 0.03 | **0.36** | **0.17** | **0.25** | **0.23** | **0.12** | **0.13** | **0.18** | **0.24** | 0.89 |  |  |  |  | 2.36 | 1.23 |
| 20. CAN Coping Motives | **0.12** | -0.08 | **-0.18** | **-0.29** | -0.02 | 0.01 | 0.02 | **0.17** | 0.01 | 0.06 | **0.14** | **0.41** | **0.15** | **0.21** | **0.20** | **0.30** | **0.43** | **0.48** | **0.38** | 0.88 |  |  |  | 2.29 | 1.27 |
| 21. CAN Enhancement Motives | 0.08 | 0.07 | -0.04 | -0.04 | 0.05 | 0.08 | 0.05 | 0.05 | 0.05 | **0.10** | **0.29** | 0.07 | **0.41** | 0.08 | **0.17** | **0.21** | **0.27** | **0.34** | **0.46** | **0.38** | 0.86 |  |  | 3.66 | 1.18 |
| 22. CAN Conformity Motives | 0.06 | **-0.08** | **-0.18** | **-0.15** | 0.07 | -0.00 | -0.00 | **0.23** | 0.07 | **0.20** | **0.20** | **0.26** | **0.19** | **0.52** | 0.01 | -0.01 | **0.17** | **0.17** | **0.36** | **0.21** | **0.10** | 0.90 |  | 1.37 | 0.74 |
| 23. CAN Expansion Motives | **0.21** | 0.01 | **-0.14** | **-0.17** | **0.13** | 0.07 | 0.09 | **0.11** | 0.02 | 0.10 | **0.20** | **0.18** | **0.23** | **0.20** | **0.18** | **0.27** | **0.29** | **0.39** | **0.40** | **0.46** | **0.41** | **0.25** | 0.92 | 2.28 | 1.30 |

Note. Significant correlations are bolded for emphasis and were determined by a 99% bias-corrected standardized bootstrapped confidence interval (based on 10,000 bootstrapped samples) that does not contain zero. Correlations are available at the OSF project website. Underlined values on the diagonal refer to Cronbach’s alphas for each variable. ALC = Alcohol; CAN = Cannabis; AUD = Alcohol Use Disorder; CUD = Cannabis Use Disorder. Typical quantity of alcohol use was calculated by summing the total number of SDUs consumed across time blocks during the typical week. Total number of SDUs consumed (summed) were transformed into grams of alcohol. We calculated typical quantity of marijuana use by summing the total number of grams consumed across time blocks during the typical week.

Table S2

*Summary of relative total, relative indirect, and relative direct effects of alcohol mediation model with low mindfulness profile as the reference group for the full analytic sample (n = 708)*

| Outcome Variable: **Past 30-day Alcohol Problems** | B | 95% CI |
| --- | --- | --- |
| Relative Direct Effects  X1 > Alcohol Problems | 0.43 | -0.38, 1.24 |
| Relative Indirect Effects  X1 > Social Motives > Alcohol Problems  X1 > Coping Motives > Alcohol Problems  X1 > Enhancement Motives > Alcohol Problems  X1 > Conformity Motives > Alcohol Problems | 0.11  **0.37**  0.04  **0.17** | -0.002, 0.27  **0.15, 0.66**  -0.10, 0.21  **0.02, 0.40** |
| Relative Total Effect  Relative Direct + Relative Indirect | **1.12** | **0.30, 1.95** |
| Outcome Variable: **Past 30-day Alcohol Problems** | B | 95% CI |
| Relative Direct Effects  X2 > Alcohol Problems  Relative Indirect Effects | **-0.92** | **-1.83, -0.01** |
| X2 > Social Motives > Alcohol Problems  X2 > Coping Motives > Alcohol Problems  X2 > Enhancement Motives > Alcohol Problems  X2 > Conformity Motives > Alcohol Problems  Relative Total Effect  Relative Direct + Relative Indirect | -0.03  **-0.18**  -0.02  **-0.10**  **-1.26** | -0.15, 0.09  **-0.38, -0.03**  -0.14, 0.06  **-0.24, -0.001**  **-2.20, -0.31** |
| Outcome Variable: **Past 30-day Alcohol Problems** | B | 95% CI |
| Relative Direct Effects |  |  |
| X3 > Alcohol Problems  Relative Indirect Effects  X3 > Social Motives > Alcohol Problems  X3 > Coping Motives > Alcohol Problems  X3 > Enhancement Motives > Alcohol Problems  X3 > Conformity Motives > Alcohol Problems  Relative Total Effect  Relative Direct + Relative Indirect | **-1.27**  -0.04  **-0.36**  0.03  **-0.20**  **-1.83** | **-2.17, -0.36**  -0.19, 0.10  **-0.59, -0.17**  -0.08, 0.17  **-0.40, -0.05**  **-2.75, -0.91** |
| Outcome Variable: **Alcohol Use Disorder Symptoms** | B | 95% CI |
| Relative Direct Effects  X1 > AUD Symptoms | 0.16 | -0.75, 1.07 |
| Relative Indirect Effects  X1 > Social Motives > AUD Symptoms  X1 > Coping Motives > AUD Symptoms  X1 > Enhancement Motives > AUD Symptoms  X1 > Conformity Motives > AUD Symptoms  Relative Total Effects  Relative Direct + Relative Indirect | 0.08  **0.50**  **0.28**  **0.21**  **1.24** | **-**0.01, 0.23  **0.23, 0.85**  **0.10, 0.54**  **0.03, 0.50**  **0.28, 2.20** |
| Outcome Variable: **Alcohol Use Disorder Symptoms** | B | 95% CI |
| Relative Direct Effects  X2 > AUD Symptoms  Relative Indirect Effects  X2 > Social Motives > AUD Symptoms  X2 > Coping Motives > AUD Symptoms  X2 > Enhancement Motives > AUD Symptoms  X2 > Conformity Motives > AUD Symptoms  Relative Total Effect  Relative Direct + Relative Indirect | -0.73  -0.02  **-0.25**  -0.15  -0.12  **-1.28** | -1.76, 0.29  -0.13, 0.07  **-0.50, -0.05**  -0.37, 0.03  -0.30, 0.001  **-2.38, -0.18** |
| Outcome Variable: **Alcohol Use Disorder Symptoms** | B | 95% CI |
| Relative Direct Effects |  |  |
| X3 > AUD Symptoms  Relative Indirect Effects  X3 > Social Motives > AUD Symptoms  X3 > Coping Motives > AUD Symptoms  X3 > Enhancement Motives > AUD Symptoms  X3 > Conformity Motives > AUD Symptoms  Relative Total Effect  Relative Direct + Relative Indirect | -0.60  -0.03  **-0.49**  **0.21**  **-0.25**  **-1.15** | -1.62, 0.41  -0.16, 0.07  **-0.78, -0.24**  **0.02, 0.47**  **-0.49, -0.06**  **-2.22, -0.08** |

*Note.* Significant effects are in bold typeface for emphasis and were determined by a 95% percentile bootstrap confidence interval (based on 10,000 bootstrap samples) that does not contain zero. For clarity, two models were conducted with past 30-day alcohol problems and AUD symptoms estimated separately as outcomes. However, for parsimony, results are presented by the profile comparison effects for each outcome. For all models, X1 = Low Mindfulness profile compared to Judgmentally Observing profile, X2 = Low Mindfulness profile compared to Non-Judgmentally Aware profile, and X3 = Low Mindfulness profile compared to High Mindfulness profile. Effects from covariates (i.e., gender and typical alcohol consumption) were not included for parsimony but are available at the OSF website.

Table S3

*Summary of relative total, relative indirect, and relative direct effects of cannabis* *mediation model with low mindfulness profile as the reference group for the full analytic sample (n = 673)*

| Outcome Variable: **Past 30-day Cannabis** **Problems** | B | 95% CI |
| --- | --- | --- |
| Relative Direct Effects  X1 > Cannabis Problems | **1.15** | **0.36, 1.94** |
| Relative Indirect Effects  X1 > Social Motives > Cannabis Problems  X1 > Coping Motives > Cannabis Problems  X1 > Enhancement Motives > Cannabis Problems  X1 > Conformity Motives > Cannabis Problems  X1 > Expansion Motives > Cannabis Problems | **-0.14**  **0.73**  **0.22**  0.11  0.09 | **-0.31, -0.01**  **0.41, 1.10**  **0.09, 0.39**  -0.02, 0.31  -0.08, 0.30 |
| Relative Total Effect  Relative Direct + Relative Indirect | **2.16** | **1.34, 2.99** |
| Outcome Variable: **Past 30-day Cannabis Problems** | B | 95% CI |
| Relative Direct Effects  X2 > Cannabis Problems  Relative Indirect Effects | **-1.86** | **-2.73, -0.99** |
| X2 > Social Motives > Cannabis Problems  X2 > Coping Motives > Cannabis Problems  X2 > Enhancement Motives > Cannabis Problems  X2 > Conformity Motives > Cannabis Problems  X2 > Expansion Motives > Cannabis Problems  Relative Total Effect  Relative Direct + Relative Indirect | 0.01  -0.16  -0.01  -0.07  -0.03  **-2.12** | -0.10, 0.11  -0.45, 0.09  -0.15, 0.13  -0.22, 0.01  -0.14, 0.03  **-3.06, -1.19** |
| Outcome Variable: **Past 30-day Cannabis Problems** | B | 95% CI |
| Relative Direct Effects |  |  |
| X3 > Cannabis Problems  Relative Indirect Effects  X3 > Social Motives > Cannabis Problems  X3 > Coping Motives > Cannabis Problems  X3 > Enhancement Motives > Cannabis Problems  X3 > Conformity Motives > Cannabis Problems  X3 > Expansion Motives > Cannabis Problems  Relative Total Effect  Relative Direct + Relative Indirect | **-1.15**  0.03  **-0.46**  **0.15**  -0.10  0.02  **-1.51** | **-2.00, -0.30**  -0.07, 0.14  **-0.74, -0.21**  **0.01, 0.32**  -0.24, 0.02  -0.04, 0.11  **-2.40, -0.62** |
| Outcome Variable: **Cannabis Use Disorder Symptoms** | B | 95% CI |
| Relative Direct Effects  X1 > CUD Symptoms | 0.79 | -0.19, 1.78 |
| Relative Indirect Effects  X1 > Social Motives > CUD Symptoms  X1 > Coping Motives > CUD Symptoms  X1 > Enhancement Motives > CUD Symptoms  X1 > Conformity Motives > CUD Symptoms  X1 > Expansion Motives > CUD Symptoms  Relative Total Effects  Relative Direct + Relative Indirect | **-0.19**  **0.97**  **0.35**  0.13  **0.35**  **2.41** | **-0.43, -0.02**  **0.56, 1.45**  **0.15, 0.59**  -0.03, 0.35  **0.12, 0.67**  **1.34, 3.48** |
| Outcome Variable: **Cannabis Use Disorder Symptoms** | B | 95% CI |
| Relative Direct Effects  X2 > CUD Symptoms | **-1.50** | **-2.59, -0.41** |
| Relative Indirect Effects  X2 > Social Motives > CUD Symptoms  X2 > Coping Motives > CUD Symptoms  X2 > Enhancement Motives > CUD Symptoms  X2 > Conformity Motives > CUD Symptoms  X2 > Expansion Motives > CUD Symptoms | 0.01  -0.21  -0.01  -0.09  -0.12 | -0.14, 0.15  -0.57, 0.13  -0.24, 0.19  -0.22, 0.03  -0.32, 0.03 |
| Relative Total Effect  Relative Direct + Relative Indirect | **-1.93** | **-3.14, -0.72** |
| Outcome Variable: **Cannabis Use Disorder Symptoms** | B | 95% CI |
| Relative Direct Effects  X3 > CUD Symptoms  Relative Indirect Effects | **-1.27** | **-2.33, -0.21** |
| X3 > Social Motives > CUD Symptoms  X3 > Coping Motives > CUD Symptoms  X3 > Enhancement Motives > CUD Symptoms  X3 > Conformity Motives > CUD Symptoms  X3 > Expansion Motives > CUD Symptoms  Relative Total Effect  Relative Direct + Relative Indirect | 0.04  **-0.61**  **0.23**  -0.13  0.08  **-1.66** | -0.09, 0.20  **-0.96, -0.29**  **0.02, 0.50**  -0.30, 0.02  -0.09, 0.28  **-2.81, -0.51** |

*Note.* Significant effects are in bold typeface for emphasis and were determined by a 95% percentile bootstrap confidence interval (based on 10,000 bootstrap samples) that does not contain zero. For clarity, two models were conducted with past 30-day cannabis problems and CUD symptoms estimated separately as outcomes. However, for parsimony, results are presented by the profile comparison effects for each outcome. For all models, X1 = Low Mindfulness profile compared to Judgmentally Observing profile, X2 = Low Mindfulness profile compared to Non-Judgmentally Aware profile, and X3 = Low Mindfulness profile compared to High Mindfulness profile. Effects from covariates (i.e., gender and typical cannabis consumption) were not included for parsimony but are available at the OSF website.
